# Loss of Gadd45b accelerates BCR-ABL-driven CML

**DOI:** 10.18632/oncotarget.26076

**Published:** 2018-09-07

**Authors:** Xiaojin Sha, Barbara Hoffman, Dan A. Liebermann

**Affiliations:** ^1^ Fels Institute for Cancer Research and Molecular Biology, Temple University, Philadelphia, PA, USA; ^2^ Department of Medical Genetics and Molecular Biochemistry, Lewis Katz School of Medicine, Temple University, Philadelphia, PA, USA

**Keywords:** Gadd45a, chronic myelogenous leukemia, stress response protein, tumor suppressor

## Abstract

Gadd45b is a member of Gadd45 stress sensor protein family that also includes Gadd45a & Gadd45g. To investigate the effect of Gadd45b in bcr-abl oncogene driven chronic myeloid leukemia (CML) development, syngeneic wild type lethally irradiated mice were reconstituted with either wild type or Gadd45b null myeloid progenitors transduced with a retroviral vector expressing BCR-ABL. Loss of Gadd45b was observed to accelerate BCR-ABL driven CML development with shortened median mouse survival time. BCR-ABL Gadd45b deficient CML progenitors exhibited increased proliferation and decreased apoptosis, associated with hyper-activation of c-Jun NH_2_-terminal kinase and Stat5. These results provide novel evidence that gadd45b, like gadd45a, functions as a suppressor of BCR-ABL driven leukemia, albeit via a different mechanism.

## INTRODUCTION

*gadd45b* (*gadd45β/MyD118*) is a member of a family of structurally and functionally related genes, that includes *gadd45a* (gadd45α/gadd45) and *gadd45g* (gadd45γ/CR6), which encode small (18kDa), evolutionarily conserved proteins, sharing high homology (55%-57%), highacidity (pI 4.0–4.2), and which are primarily localized to the nucleus [1–3]. *gadd45b* and other gadd45 genes are essentially expressed in all tissues and function as DNA damage sensors. Gadd45 proteins are involved in cell cycle regulation, DNA replication and repair through associating with protein partners, including p21, a cyclin-dependent kinase inhibitor, cdc2/cyclinB, a key kinase for G2/M progression and proliferating cell nuclear antigen (PCNA), and the p38 and JNK stress-response kinases [1].

Gadd45b plays an important role in apoptosis and survival. Blocking Gadd45b by antisense expression in M1 myeloblastic leukemia cells impaired TGFb-induced cell death [4]. Yoo et al demonstrated that TGFb-induced apoptosis was blocked in hepatocytes from Gadd45b^-/-^ mice [5]. Ectopic expression of Gadd45b enhanced stress mediated apoptosis in both M1 leukemia and H1299 lung carcinoma cells [6]. Although gadd45b seems infrequently mutated in cancer, reduced expression of Gadd45b gene due to promoter methylation was observed in hepatocellular carcinoma [2]. These evidences indicate that gadd45b is a pro-apoptotic protein. Nevertheless, in another setting, Gupta et al found that Gadd45b protected hematopoietic cells from UV-induced apoptosis [7–8], suggesting that Gadd45b apoptoic/survival functions are context dependent. NIH3T3 cells overexpressing Gadd45b form tumors in NOD/SCID mice [9].

The Philadelphia chromosome (Ph) arises from a balanced translocation involving chromosomes 9 and 22 [10]. This translocation forms the fusion protein BCR-ABL which is essential for cell transformation due to its constitutive tyrosine kinase activity. 90% of CML patients are Ph positive. Chronic myelocytic leukemia (CML) is a myeloproliferative disorder of hematopoietic stem cells and progenitors. It is characterized by progression from an indolent ‘chronic phase’ (CML-CP), a phase in which mature granulocytes hyperproliferate, to the aggressive and fatal ‘blast crisis’ (CML-BC) marked by the clonal expansion of differentiation-arrested immature blasts [11–13]. Importantly, Gadd45a was found to supress BCR-ABL driven CML in a mouse model (14). Clearly, it was of interest to examine the possible role of Gadd45b in modulating BCR-ABL driven leukemia. The present study indicates that loss of Gadd45b, like Gadd45a, accelerated CML, behaving as a tumor suppressor, albeit by a different mechanism than Gadd45a.

## RESULTS

### Loss of Gadd45b accelerates BCR-ABL-driven leukemia development

**Figure 1:**
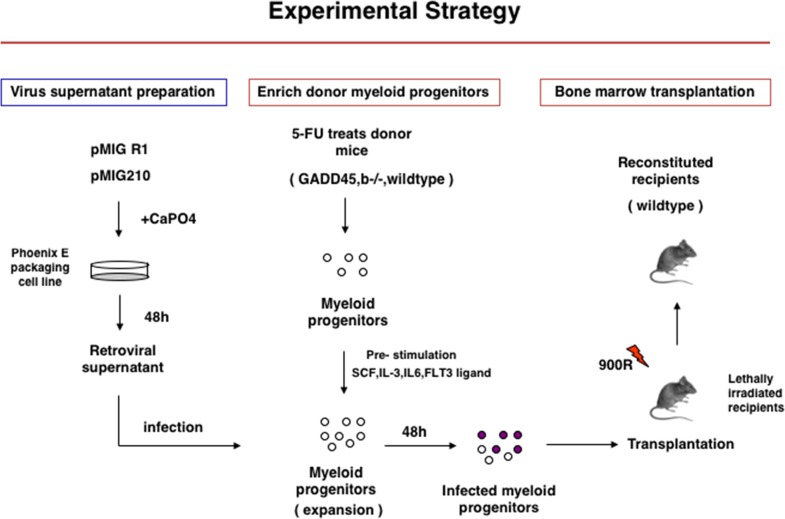
Experimental Startegy of Gadd45b^-/-^ and wild type bone marrow transplantation into syngenic sub-lethaly irradiated mice

**Figure 2:**
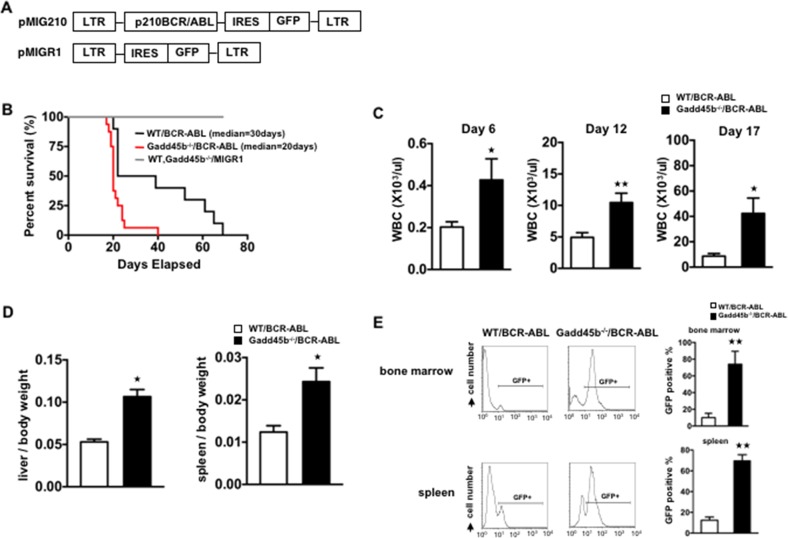
Loss of gadd45b accelerated the development of leukemia in Gadd45b^-/-^/BCR-ABL recipients **(A)** The retroviral vector expressing BCR-ABL oncoprotein and GFP reporter protein. **(B)** Gadd45b^-/-^/BCR-ABL recipients showed accelerated onset of leukemia. Kaplan Meier survival curve of wild type recipients transplanted with equivalent numbers of *bcr-abl* transduced bone marrow cells from either 5-FU treated Gadd45b^-/-^ or wild type donors. The median survival of Gadd45b^-/-^/BCR-ABL recipients was 20 days (n=16) compared to the WT/BCR-ABL recipients that was 30 days (n=10, *p* < 0.05). Recipients of control vector transduced BM cells from 5-FU treated Gadd45b^-/-^ and wild type donors did not develop leukemia (n=7 for each genotype). Results were derived from three independent experiments. **(C)** Iincreased peripheral blood WBC in Gadd45b^-/-^/BCR-ABL recipients compared to WT/BCR-ABL recipients. Data were from wild type recipients at 6 days, 12 days and 17 days post BM transplantation (^**^ *p* < 0.01, ^*^ *p* < 0.05, error bar represents SEM, each genotype n=6-7). **(D)** Enlarged spleen and liver in Gadd45b^-/-^/BCR-ABL recipients compared to WT/BCR-ABL recipients. Bar graphs show the ratio of spleen and liver weights to body weights. (^*^ *p* < 0.05, error bar represents SEM, n=5-6). **(E)** Increased GFP positive cells in BM and spleen of Gadd45b^-/-^/BCR-ABL recipients compared to WT/BCR-ABL recipients using flow cytometry analysis. Bar Graph shows percentage of GFP+ cells (^**^ *p* < 0.01, error bar represents SEM, n=5). Data of D and E were from wild type recipients sacrificed 20 days post BM transplantation.

To address the possible role of Gadd45b in modulating bcr-abl induced leukemia, myeloid progenitor enriched bone marrow cells were isolated from both wild type and Gadd45b^-/-^ mice and transduced with either control MSCV-IRES-EGFP, also termed as MIGR1, retrovirus vector or MIG-BCR-ABL encoding vector (Figure 1 Figure 2A). Two days after infection, 5000 GFP positive cells mixed with 495,000 GFP negative accessory cells were retroorbitally transplanted into γ-irradiated wild type syngeneic recipient mouse. As shown in Figure 2B, survival of recipient mice that were transplanted with MIG-BCR-ABL Gadd45b^-/-^ myeloid progenitors was significantly curtailed compared to mice transplanted with MIG-BCR-ABL wild-type progenitors, due to accelerated development of CML like disease, as manifested by increased WBC (Figure 2C), increased liver weight (Figure 2D), and increase in GFP + cells in the bone marrow and spleen (Figure 2E).

### Loss of Gadd45b does not block the differentiation of myeloid progenitors in Gadd45b^-/-^/BCR-ABL recipients

**Figure 3:**
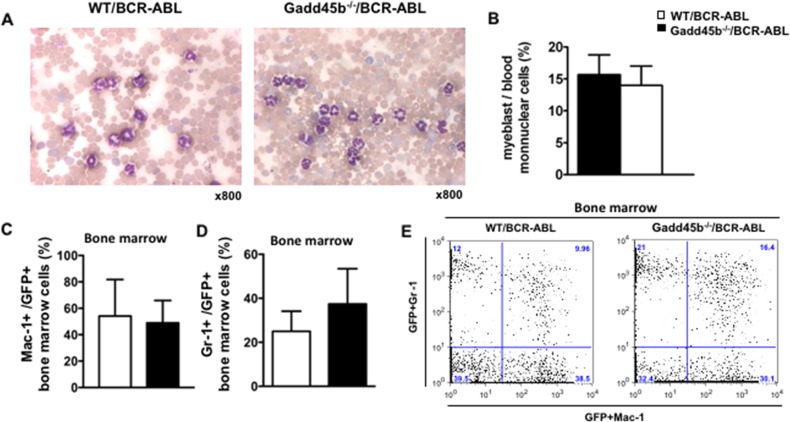
Both Gadd45a^-/-^/BCR-ABL and WT/BCR-ABL recipients developed CML **(A)** Blood smear from moribund recipients. The majority of mononuclear cells from both WT/BCR-ABL recipients (left) and Gadd45b^-/-^/BCR-ABL recipients (right) were mature myeloid cells. (Wright-Giemsa staining, magnification x800, n=6-7). **(B)** Myeloblasts count from peripheral blood. Myeloblast percentiles in the peripheral blood of moribund Gadd45b^-/-^/BCR-ABL recipients were similar as that of moribund WT/BCR-ABL recipients at around 15%. 200 mononuclear cells were counted for each sample (error bar represents SEM, n=6). **(C)**. **(D)** Similar percentiles of myeloid lineage markers Mac-1 and Gr-1 expression cells in GFP positive BM cells of both moribund genotype recipients (error bars represent SEM, n=5-7). **(E)** A representative BM dot blot flow cytometry analysis picture shows Mac-1 and Gr-1 expression in both genotypes recipients.

To address whether loss of Gadd45b affected the differentiation of myeloid progenitors, myeloblasts were counted from the blood smears obtained from moribund recipients. As shown in Figure 3A, 2B, the majority of mononuclear cells from both WT/BCR-ABL and Gadd45b^-/-^BCR-ABL recipients were mature myeloid cells. Bone marrow cells were harvested from moribund recipients and analyzed for the expression of GFP and myeloid lineage differentiation cell-surface markers Mac-1 and Gr-1. It was observed that gadd45b^-/-^ leukemic cells exhibited similar levels of Mac-1 and Gr-1 as wild type leukemic cells (Figure 3C, 2D, 3E). These results indicate that acceleration of CML due to loss of Gadd45b is not associated with blocked differentiation.

### Loss of Gadd45b causes enhanced proliferation and decreased apoptosis of myeloid progenitors

**Figure 4:**
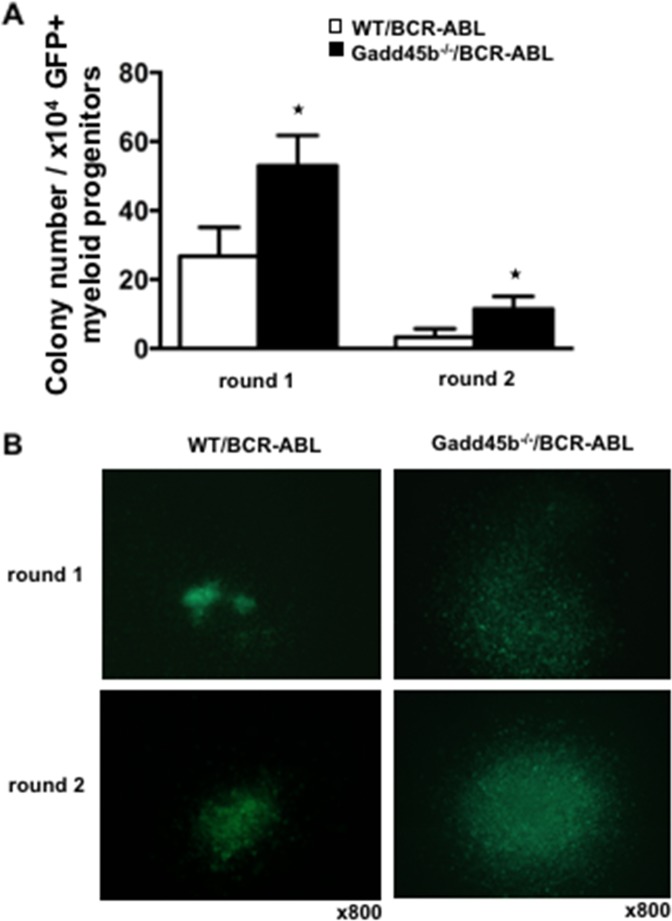
Loss of Gadd45b increased colony forming units (CFU) *in vitro* **(A)** Increased colony forming units (CFU) per round of Gadd45b^-/-^/BCR-ABL myeloid progenitors in serial replating assay (^*^ p < 0.05, each genotype n=4). **(B)** Representative pictures of colonies of Gadd45b^-/-^/BCR-ABL (right panel) were larger than WT/BCR-ABL myeloid colonies (left panel). Magnification x 800.

**Figure 5:**
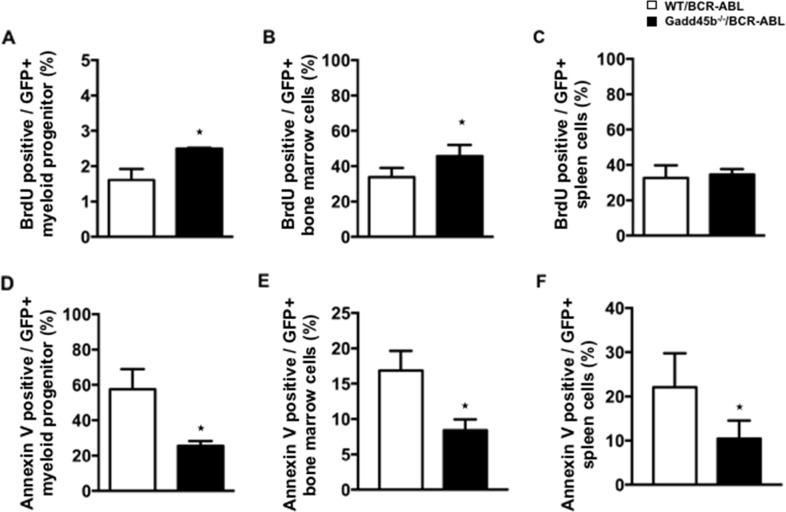
Loss of Gadd45b reduced apoptosis of myeloid progenitors in Gadd45b^-/-^/BCR-ABL recipients **(A, B, C)** Wild type recipients 20 days post transplantation with either WT/BCR-ABL or Gadd45-/-/BCR-ABLBCR bone marrow were euthanized; myeloid progenitors (A, D), were cultured for 2 weeks, whereas bone marrow (B, E) and spleens (C, F) were analyzed following isolation. Enhanced proliferation capacity was observed in Gadd45b^-/-^/BCR-ABL myeloid progenitors *in vitro* but not in either BM or spleen of Gadd45b^-/-^/BCR-ABL myeloid progenitor recipients compared to WT/BCR-ABL recipients, determined by BrdU assay. (^*^ *p* < 0.05, error bar represents SEM, each genotype n= 4-5). **(D, E, F)** Decreased Annexin V positive apoptotic cells were observed not only in Gadd45b^-/-^/BCR-ABL myeloid progenitors *in vitro* but also in the BM and spleen of Gadd45b^-/-^/BCR-ABL myeloid progenitor recipients compared to WT/BCR-ABL recipients(^*^ *p* < 0.05, error bar represents SEM, each genotype n=3-5).

Using successive rounds of a colony forming assay in semisolid methylcellulose, it was observed that Gadd45b^-/-^/BCR-ABL myeloid progenitors formed significantly more and larger colonies than WT/BCR-ABL myeloid progenitors (Figure 4A, 4B). To address whether Gadd45b deficiency affected the intrinsic proliferation capacity of myeloid progenitors, the same number of BCR-ABL- transduced wild type and Gadd45b^-/-^ primary bone marrow myeloid progenitors were cultured for 2 weeks and prior to BrdU assay As shown in Figure 5A, 5B the percentage of BrdU-positive cells in GFP positive Gadd45b^-/-^/BCR-ABL progenitor and bone-marrow cells was significantly higher than in GFP positive WT/BCR-ABL cells. Collectively these data suggest that loss of Gadd45b enhances the proliferation capacity of myeloid progenitor.

Next we examined the percentage of apoptotic cells using Annexin V staining and flow cytometry analysis. Decreased proportion of Annexin V+GFP+ cells were detected in Gadd45b^-/-^/BCR-ABL myeloid progenitors compared to WT/BCR-ABL myeloid progenitors. The proportion of Annexin V+GFP+ bone marrow cells and spleen cells were also significantly decreased in Gadd45b^-/-^/BCR-ABL recipients compared to WT/BCR-ABL recipients (Figure 5D, 4E, 4F), indicating that loss of Gadd45b decreases apoptosis of BCR-ABL expressing myeloid progenitors.

Taken together, these data suggest that acceleration of CML in mice transplanted with MIG-BCR-ABL Gadd45b^-/-^ progenitors compared to mice transplanted with wild type MIG-BCR-ABL progenitors is due to enhanced proliferation and decreased apoptosis of CML progenitors.

### Loss of Gadd45b hyperactivates c-Jun NH_2_-terminal kinase (JNK) and Stat5: The phosphorylation of p38MAPK and AKT in GFP+Gadd45b^-/-^/BCR-ABL myeloid progenitors was similar as GFP+WT/BCR-ABL

**Figure 6:**
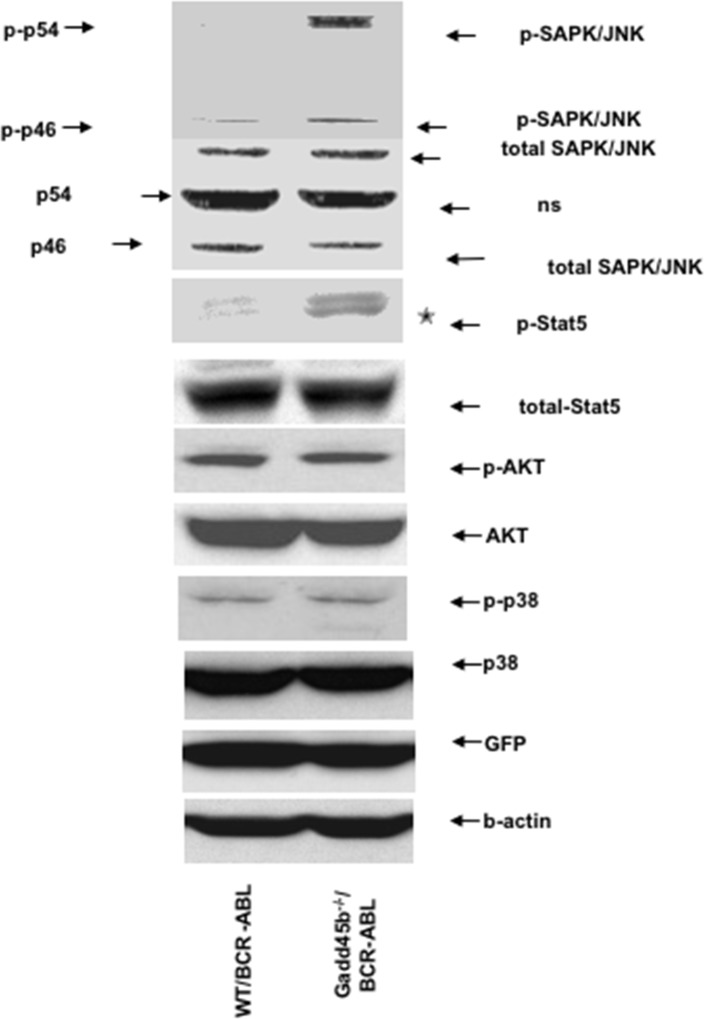
Loss of Gadd45b hyperactivates JNK and Stat5 Hyperphosphorylation of JNK and Stat5 in sorted GFP+ Gadd45b-/-/BCR-ABL myeloid progenitors (2 weeks culture) compared to GFP+ WT/BCR-ABL myeloid progenitors *in vitro*. The phosphorylation levels of AKT and p38 in sorted GFP positive Gadd45b-/-/BCR-ABL myeloid progenitors were similar as WT/BCR-ABL myeloid progenitors *in vitro*. The experiments were repeated 3 times independently.

BCR-ABL activates multiple signaling pathways including p38MAPK, PI3K/AKT, JNK, STAT5 through its deregulated tyrosine kinase activity to promote proliferation and block differentiation. Our previous study showed that loss of Gadd45a hyperactivated p38MAPK, PI3K/AKT and STAT5 signaling pathways to promote proliferation and reduce apoptosis in BCR-ABL transformed myeloid progenitors [14]. Thus, it was of interest to determine if the CML suppressive function of Gadd45b is mediated via similar or different signaling pathways. As shown in Figure 6, JNK (p-p54) was observed to be phosphorylated (activated) in GFP positive Gadd45b^-/-^/BCR-ABL myeloid progenitors cells but not in GFP positive WT/BCR-ABL myeloid progenitors. Furthermore, GFP positive Gadd45b^-/-^/BCR-ABL myeloid progenitors exhibited significantly increased phosphorylation of Stat5 compared to WT/BCR-ABL cells. However, the phosphorylation levels of p38MAPK and AKT in BCR-ABL transduced Gaddd45b deficient myeloid progenitors was similar to their phosphorylation status in BCR-ABL transduced wild type controls.

## DISCUSSION

Using BCR-ABL-induced CML-like myelo-proliferative disease mouse model, it was observed that loss of Gadd45b accelerated CML progression and reduced survival of mice. Leukemic progenitors that lack Gadd45b expression were not blocked in differentiation; however, they exhibited increased proliferation and reduced apoptosis. Furthermore, loss of Gadd45b expression appeared to result in constitutive activation of JNK and STAT5.

Collectively, these result indicate that the CML suppressive phenotype of Gadd45b differs from the one observed for Gadd45a, where loss of Gadd45a resulted in increase in AKT and p38 activation but not JNK. Thus, it will be of interest to determine the consequence of loss of both Gadd45a and gadd45b on CML progression. Along similar lines, it will be interesting to explore if/how loss of Gadd45g modulates the CML phenotype.

We have also observed that Gadd45a expression in samples obtained from CML patients was upregulated in more indolent chronic phase CML samples and down regulated in aggressive accelerated phase CML and blast crisis CML [14]. Thus, studies are underway to determine the expression status of Gadd45b in CML patients.

## MATERIALS AND METHODS

### Mice and genotyping

Gadd45b^−/−^ mice (in a C57BL/6 × 129Sv background) were graciously provided by Albert Fornace (Harvard University, Boston, MA, USA) and kept in specific pathogen-free animal facilities at Medical School of Temple University. Mice were genotyped by PCR. PCRs using three primers allowed for simultaneous detection of the WT and mutant Gadd45b allele. These primers consisted of a 5′ upstream primer (5′-GCTGTGGAGCCAGGAGCAGCA −3′), a common 3′ downstream primer (5′-ATATGCAAGCGATCTGTCTTGCTCA-3′), and a neo-specific primer (5′-AAGCGCATGCTCCAGACTGCCTT-3′). Reactions were run for 37 cycles of 94°C for 1 minute, 63°C for 14 seconds, and 72° for 12 seconds. All animal studies were approved by Temple University Institutional animal use and care committee.

### Cell culture

Myeloid progenitors which were isolated from the bone marrow of 5-fluorouracil (5-FU, 150mg/kg) treated mice were cultured in StemPro-34 SFM complete Medium (GIBCO) supplemented with cytokine cocktail SCF (100ng/ml), IL-3 (20ng/ml), IL-6 (20ng/ml), Flt-3 (100ng/ml) before infection. For *in vitro* molecular signaling detection, the concentration of the cytokines were reduced as follows: SCF (12ng/ml), IL-3 (5ng/ml), IL-6 (5ng/ml). Cells were maintained in a humidified atmosphere with 5% CO2 at 37°C.

### BCR-ABL-expressing retrovirus vector and retroviral transduction

The retroviral vector MSCV-BCR/ABL-IRES-GFP and retroviral transduction of mouse bone marrow derived myeloid progenitors for induction of CML by BCR-ABL has been described previously [13]. MSCV-IRES-GFP (MIGR1) and MIGR1-BCR-ABL vectors were the gift from Warren Pear. Phoenix E packaging cells (Orbigen, Inc) were transfected with either MIGR1 or MIGR1-BCR-ABL using calcium phosphate precipitation method. 48 hours after transfection, the retroviral supernatants were harvested and centrifuged at 3200rpm for 10 minutes at 32°C. Finally, the supernatant was frozen at −80°C. To infect the enriched myeloid progenitors which were from the bone marrow of 5-fluorouracil (5-FU, 150mg/kg) treated WT and Gadd45b^-/-^ mice 5 days in advance, 3 ml retroviral supernatant supplemented with 10μg/ml polybrene (Sigma-Aldrich) was added to 1×10^6^ cells and centrifuged at 2200rpm for 45 minutes at 32°C. Spinoculation was repeated 4 times within 2 days. The infection efficiency was determined on the basis of green fluorescence by flowcytometry 24 hours after the fourth round infection.

### Bone marrow transplantation

The syngeneic wild type recipient mice (6-12 weeks old) were lethally irradiated with 900 rads (^137^Cs source). 5×10^3^ GFP positive either wild type or Gadd45b^-/-^ myeloid progenitors along with 495,000 GFP negative accessory cells were introduced into each wild type recipient by retro-obital injection.

### FACS analysis and cell sorting

Hematopoietic cells were collected from the bone marrow and spleen of mice. Erythrocytes were lysed in NH_4_Cl red blood cell lysis buffer (pH 7.4). The cells were washed with PBS and stained with myeloid lineage marker Mac-1and Gr-1. After staining, the cells were washed once with PBS and subjected to FACS analysis. Cells were analysed with FACScalibur. Antibodies were obtained from BD Pharmingen. Influx cell sorter (Becton Dickinson) was used for sorting GFP positive cells.

### Colony forming assay

48hour-post infected 2×10^4^ GFP positive myeloid progenitors were plated in semisolid methylcellulose (MethoCult M3534, StemCell Technologies) into 35-mm^2^ Petri dishes and were incubated at 37°C in humidified atmosphere of 5% CO_2_ in air. The GFP positive colonies were scored and harvested in 7 days. 2×10^4^ GFP positive cells were plated again for the 2^nd^ round.

### Apoptosis

The percentage of apoptotic GFP positive cultured myeloid progenitors, hematopoietic cells from bone marrow and spleen of the 20 days post transplantation wild type recipients were analysed with FACSCalibur after staining cells with the Annexin V-APC Apoptosis Detection Kit (eBioscience) according to manufacturer’s protocol. Flowjo 9.9.4 software (Tree Star) was used for data analysis.

### BrdU assay

The BrdU incorporate assay was performed using BrdU-APC flow kit according to the manufacturer instruction (BD Pharmingen). For *in vitro* experiment, the BCR-ABL- transducted primary bone marrow myeloid progenitors were cultured for 2 weeks. 20ul of BrdU solution directly added to 2ml medium that contained 6×10^5^ cells and incubated for 35 minutes. For *in vivo* experiment, 20 days post transplantation wild type recipients were intraperitoneally injected with 100ug/g BrdU solution (10mg/ml). 24 hours post injection, bone marrow and spleen cells were extracted from mice. The cultured progenitors, bone marrow and spleen cells were finally subjected to FACS analysis using FACSCalibur. Flowjo 9.9.4 software (Tree Star) was used for data analysis.

### Histology and cytology

Tissues fixed in 10% buffered formalin were embedded in paraffin and sectioned. The sections were stained with hematoxylin and eosin (University of Pennsylvania Core Histologic Facility, Phiadelphia, PA, USA). Blood smears were stained in May-Gruenwald-Giemsa (GIBCO).

### Western blotting

Total protein lysates were prepared by resuspending bone marrow cell pellets at a concentration of 10^7^/ml in CST-cell lysis buffer (Cell Signaling technologies, MA, USA). Protein concentration was determined using Bio-Rad protein assay (Bio-Rad) followed by spectrophotometer readout at a wavelength of 595-nm. Sixty micrograms of each sample was fractionated on SDS-PAGE gels and the expression of protein were detected by Western blotting with specific antibodies. We used the following antibodies: antibodies from Cell signaling Technology include phospho-Akt (Ser473) (#9271), AKT (#9272), Phospho-SAPK/JNK (Thr183/Tyr185) (#9251), SAPK/JNK(#9252), Phospho-p38 MAPK (Thr180/Tyr182) (3D7) (#9215), p38 MAPK (#9212), phosphor-Stat5 (#9359), Stat5 (#9363), β-Actin (#4967), GFP(#2555).

### Statistical analysis

Statistical analysis was performed by using student t test (^*^: *p* < 0.05, ^**^: p < 0.01) (GraphPad Prism Software, San Diego, CA, USA).
